# School-based Psychosocial interventions on mental health among Chinese rural children with traumatic experiences: a protocol using cluster randomized controlled trial

**DOI:** 10.1186/s40359-023-01182-7

**Published:** 2023-05-04

**Authors:** Jing Guo, Xiaohan Liu, Ning Huang, Fan Yang, Yashuang Bai, Bo Zhang, Paul Lodder

**Affiliations:** 1 Department of Health Policy and Management, School of Public Health, Peking University, 38 Xueyuan Road, Beijing, 100191 P. R. China; 2 Department of Neurology and ICCTR Biostatistics and Research Design Center, Boston Children’s Hospital, Harvard Medical School, Boston, MA 02115 USA; 3 Department of Methodology and Statistics, Tilburg University, Tilburg, The Netherlands

**Keywords:** Mental Health, Traumatic experiences, Rural children, School-based

## Abstract

**Background:**

The first aim of this study is to test the effectiveness of school-based psychosocial interventions for improving mental health in rural Chinese children with traumatic experiences. The second aim is to examine which individual, family and school related factors could explain the effectiveness of school-based psychosocial interventions. Third, we will investigate whether individual, family, and school related conditions play a moderator role on the effectiveness of school-based psychosocial interventions.

**Methods:**

This study will conduct a cluster randomized controlled trial (RCT) in a large sample of Chinese rural children. Four rural counties in Shandong (Central China), Henan (Central China), Inner Mongolia (Northern China), and Xinjiang (Western China) will be selected as study settings from which schools will be sampled. Each sampled school will be randomly allocated either the intervention groups or a control group. Randomization will be performed by the research member who is not involved in the intervention stage. In each school students in grade 5 or higher will be recruited to ensure that approximately 50 children aged 10 to 18 years will be included. In each county, one high school, one middle school, and one primary school will be randomly chosen as the intervention group, and the other three similar schools will be chosen as control (waiting list) groups. A standardized and uniform research protocol will be applied in all intervention schools. All school social workers and psychological teachers would receive one week of in-person training following procedures. School-based psychosocial interventions included 14 group sessions for 14 consecutive weeks.

**Discussion:**

This study would develop school-based mental health promotion policy recommendations to improve Chinese rural children’s mental health. This study can provide solid evidence for the promotion of school-based intervention in general.

**Trial registration:**

ChiCTR2300069405, Registered on 15 March 2023.

## Background

Trauma survivors are at high risk of many adverse mental health outcomes, most prominently posttraumatic stress symptoms(PTSS), self-injurious behavior, and other psychotic symptoms [1, 2]. Experiencing trauma in childhood—a crucial period for the developing brain and personality—is a key risk factor for developing psychopathology later in life [1]. Chinese families and governments have suffered enormous economic and social losses from the negative consequences of childhood trauma. One study estimated that emotional and physical abuse in childhood accounts for 26.3% and 12.2% of the disability-adjusted life-years (DALYs) lost because of mental disorders [3]. China lost 0.84% of Gross Domestic Product (GDP) due to child physical abuse and 0.47% and 0.39% of GDP due to child emotional abuse and child sexual abuse, respectively [3]. These findings indicate that it is necessary to establish beneficial interventions to reduce the likelihood of developing psychopathology among victimized children. Meta-analyses of worldwide samples show self-reported rates of 22.6% for physical abuse, 16.3% for physical neglect, and 18.4% for emotional neglect [4]. In China, a recent meta-analysis revealed that the pooled prevalence of child physical abuse, physical neglect, and emotional neglect were 20%, 47%, and 44%, respectively [2]. Thus, it is necessary to identify and conduct intervention programs for improving mental health status among Chinese victimized children.

### Effectiveness of school-based psychosocial interventions on mental health among children

Schools are a safe environment and can provide supportive trainings and interventions to universal children. School-based psychosocial interventions for traumatic psychological usually involve three types of interventions: preventive interventions in school or classroom level, universal psychosocial support interventions, and targeted trauma-focused cognitive behavioral therapy (TF-CBT) [5]. In particular, trauma-focused CBT has been proven to be an effective treatment of many commonly experienced mental problems by traumatized children, such as PTSD, depression [6]. Although TF-CBT approach is increasingly recommended, few studies have evaluated the effectiveness of multilayered interventions. In addition, the effectiveness of these interventions relies on not only the intervention content but also implementers. The worldwide shortage of mental health professionals limits the development of primary mental health services. In China, child mental health human resources are scarce, with fewer than 500 full-time child psychiatrists in the whole country [7]. Especially unique to Chinese children are the salient mental health disparities between urban and rural areas, and rural children experience more childhood trauma [2, 8]. Moreover, child psychiatrists are unequally distributed in China, as most of them are concentrated in major cities such as Beijing, while in rural areas, there are no specialist child psychiatry services [7]. Therefore, cluster randomized clinical trials in school settings by school social workers and headteachers may be a cost-effective and feasible way to improve rural children’s mental health.

The results of previous school-based intervention studies seem inconsistent and lack statistical power. One study suggests that a multilayered psychosocial care package seems feasible and satisfactory in reaching out to substantial populations of distressed children through different levels of care [9]. Another study found that the school-based psychosocial intervention provided by paraprofessionals helps maintain hope and reduce PTSD symptoms in children affected by political violence [10]. However, other results suggest that the comprehensive school-based intervention model was effective, although different service components affected different student outcomes. One study conducted among refugee youths reported that students across all tiers of the program demonstrated improvements in mental health and resources. However, the sample size per tier was small, and comparative analyses between tiers were not possible to conduct [11]. In addition, few studies that have tested the effectiveness of school-based psychosocial interventions among Chinese children [12] are based on small sample sizes and short-term follow-ups, limiting the validity and generalizability of the results. In this study, we will perform a classroom-based, preventive intervention that provides tools to create a safe classroom environment for children.

### Mechanism of psychosocial interventions at multiple levels

Strong evidence was found for several mechanisms explaining the effectiveness of psychosocial interventions to improve mental health among traumatized children. The first mechanism improves children’s capacities, including resilience, improving unfavorable personality traits, effective coping style, and mastery of traumatic experiences. Resilience is a crucial aspect in the child psychopathology area [13]. Some studies have shown that higher levels of resilience are related to fewer depressive and/or anxiety symptoms [14, 15]. Extensive evidence from a longitudinal study confirmed that resilience reduced the risk of depressive symptoms in individuals with childhood trauma experience [16]. In addition, personality traits reflecting stress control and self-regulation abilities are also important [17]. A study found that children with adaptive personality traits were liable to experience positive emotions, obtain social interaction and adopt an effective problem-solving method when encountering adversities [15]. Moreover, growing evidence has begun to show that factors related to children’s capacities, such as coping ability, might offer new psychosocial interventions to prevent and improve children’s mental health [18].

The second mechanism of effective psychosocial interventions in children is enhancing family capacity, which involves support for parents’ and caregivers’ ability through psychoeducation and communication skills. Parents are usually the ones who interact the most with children and adolescents. Offering group interventions to parents may be an effective way to support them and their children by sharing experiences in a similar situation [19] and learning how to support their child in managing psychological symptoms [20–22]. The World Health Organization (WHO) advises parents to communicate with their children in an honest and age-appropriate way that addresses children’s concerns and eases their anxiety. In this way, close and open communication between parents and children may serve as a protective factor in children’s mental health [23]. Research suggests that parent‒child discussions could change individuals’ perceptions and cultivate positive mindsets, which in turn reduce children’s depressive, anxiety, and stress symptoms [24]. Therefore, interventions to enhance family capacity, such as parent‒child communication skills, could help traumatized children cope with mental health problems.

The third mechanism is strengthening family relationships, which is especially important in facing traumatic events. A growing body of evidence highlights the important role of families in the prevention of internalizing problems, of which family relationships were the most frequently reported to be associated with mental health symptoms among children and adolescents [25, 26]. Zheng Ren’s study selected middle school students as participants and found that poor parent‒child relationships were associated with an increased risk of depressive symptoms [27]. Additionally, poor parental relationships may expose children to negative feelings about traumatic events and worse their mental health symptoms [27]. Thus, given the direct and indirect effect of family relationships on mental health problems among children, urgent consideration should be given to facilitate better family relationships, especially when facing traumatic events.

The fourth mechanism is providing a supportive environment, which is indicated by trusting relationships with therapists, teachers, and others. In schools, teaching social, emotional and behavioral control skills contributes to the development of personal relationships among students, as they provide the opportunity for students to discuss difficult feelings in a safe and supportive environment [28]. Adolescents face important interpersonal challenges, including the renegotiation of relationships with parents and increased involvement with peers and friends [29]. According to research findings, a supportive relationship between children and teachers is associated with a reduction in aggressive behavior [30] and can contribute to preventing behavior problems and enhancing students’ psychosocial development and adaptation [28]. Negative relationships with teachers constitute a predictive factor of the onset of psychiatric disorders and school failure [31]. In addition, teachers more actively promote mental health in the presence of positive personal relationships [32]. Therefore, more attention should be given to recruiting and preparing teachers or therapists capable of establishing trusting and supportive relationships with traumatized children.

Thus, conducting school-based psychosocial interventions from the above four perspectives are feasible and effectiveness methods. First, we will provide mental health knowledge and communication skills for children to encourage them talk their difficulties to parents, school counselor, or health care provider. Second, we plan to introduce the child mental health symptoms to parents, so that they can understand their children’s problem. Third, emotional expression and communication skills training will be employed to enhance family relationship. Forth, series training will be set up to build a supportive environment, which will help teachers to identify mental health risk and conduct regular psychological counseling for students.

## Aims and hypotheses

Based on the evidence and research gaps presented above, the aim of the current study is to explore the effectiveness of school-based psychosocial interventions on PTSS and other mental health statuses in Chinese rural children who experienced a traumatic event. To achieve this aim, we will conduct a cluster RCT in a large sample of Chinese rural children. The criteria for intervention effectiveness were reducing mental health problems through increasing positive coping style or resilience compared to children in waiting-list schools. The second aim is to examine which school-related and individual factors could explain and affect the effectiveness of school-based psychosocial interventions. Third, we analyze whether individual, family, and school-related conditions moderator the effectiveness of school-based psychosocial interventions. Specifically, we answer the following study questions and test the corresponding hypothesis:

1. Do school-based psychosocial interventions have a positive effect on children’s mental health? (a) We hypothesize that posttraumatic stress symptoms will decrease significantly among children in intervention schools but not in control schools. (b) We hypothesize that other mental health problems, such as depressive symptoms, anxiety symptoms, and bullying behaviors, will decrease significantly among children in intervention schools but not in control schools.
2. Do multilayered related factors have a mediating effect on children’s mental health? We hypothesize that the participation of students, parents, school social workers and headteachers in the psychosocial interventions increases their sense of self-efficacy, communication skills, and decreases their stress, which in turn are associated with decreased mental health symptoms among the children in the intervention schools.
3. Do multilayered related factors moderate the psychosocial intervention effect on children’s mental health? We hypothesize that the individual, family and school social workers’ and headteachers’ characteristics and the school’s environment are associated with stronger changes in the relationship between positive coping, resilience and mental health symptoms among the children in the intervention schools.

### Study significance

Based on a solid foundation of child mental health studies, this study will answer key questions regarding the effectiveness of school-based psychosocial interventions for improving mental health in rural Chinese children. This study will apply an implementation science approach, and we expect to develop a cultural adaptation of intervention strategies in the Chinese cultural context. The direct translational research-to-policy implications of this work can revolutionize how mental health intervention programs are delivered in rural primary, middle and high schools.

## Methods

### Study design and randomization

**Fig. 1:**
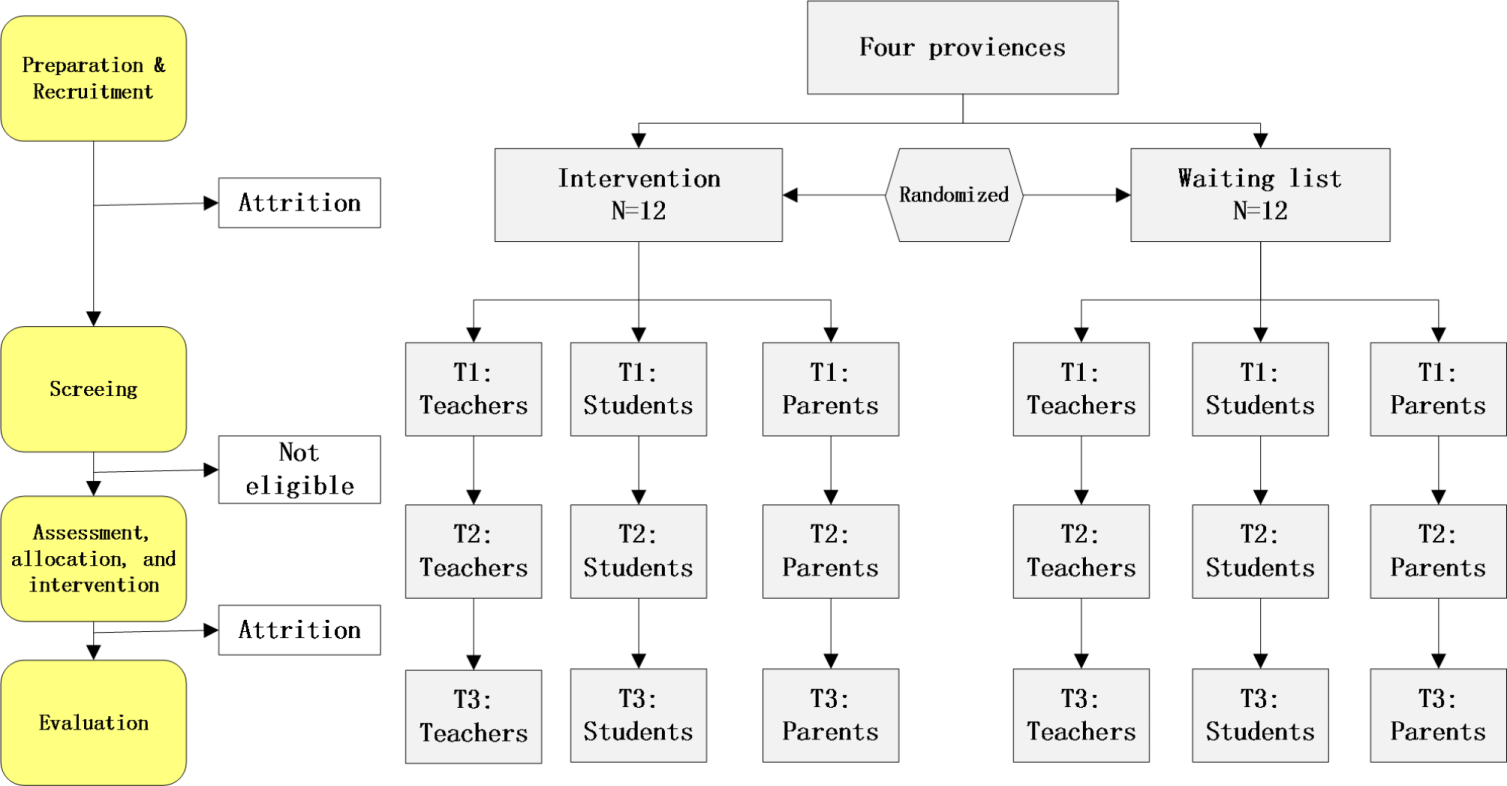
Flowchart of intervention and control situations in the study design

As shown in Fig. 1, this study is a multicenter cluster randomized controlled trial. School as the study unit will be randomly assigned to the intervention or the control group. Four rural counties in Shandong (Central China), Henan (Central China), Inner Mongolia (Northern China), and Xinjiang (Western China) will be selected as study settings. These counties were selected due to their vulnerability in socioeconomic development and weakness of the mental health system. In addition, we have good cooperation with the local schools in these areas, which ensure a high participation rate. The schools were randomly allocated to either the intervention or control group using a random number generator. Randomization will be performed by the research member who is not involved in the intervention stage. Grade 5 above in each school will be recruited to ensure that approximately 50 children aged 10 to 18 years are included. After being informed about the survey’s aims, children will be asked to provide informed consent and informed that they can join the study voluntarily. Informed consent also will be obtained from their parents/legally authorized representatives.

### Sample sizes and subjects

The required sample size to detect an expected intervention effect with sufficient statistical power was calculated for the primary outcome posttraumatic stress symptoms, as measured with the PTSD Checklist for DSM-5 (PCL-5) scale, using a method that considers the estimation of effect size, repeated measurements, significance level, power, allocation ratio, and attrition rate at T2, and T3. The following parameters are used: (1) the effect size estimations for the primary outcome is 0.20 (based on a meta-analysis [33]); (2) the intraclass correlation is 0.05; 4) 5% level of significance; 5) 80% level of power; 6) allocation ratio of 1:1; and 7) attrition rate of 20%. Under these hypotheses, we expect a sample size of at least 24 clusters (i.e., schools) with 50 children in each school to be included, resulting in a total intended sample size of 1200 children, 1200 parents and 30 teachers. The sample size will be distributed as evenly as possible among the control schools and intervention schools.

Eligibility criteria: As school is our intervention unit, the inclusion criterion is that the school should not have a similar psychosocial intervention program. For students, all children in the program schools who have signed informed consent will participate in this program. Exclusion criteria: Children with severe physical and mental health problem, and unable to participate into the program completely.

### Interventions

A standardized and uniform research protocol was applied in all intervention schools. Schools in the intervention group will participate in all intervention modules listed in Table 1. All school social workers and psychological teachers would receive one week of in-person training following procedures. School-based psychosocial interventions included 14 group sessions for 16 consecutive weeks. Child groups and guardian groups met concurrently, with joint child guardian activities in some sessions. Child group sessions were led by 1 social worker and 1 headteacher, and guardian group sessions were led by 1 social worker, with social workers randomly assigned to groups to allow examination of social worker-level effects. Schools randomized to the waiting-list control group will provide usual care throughout the follow-up time, including services to which children had access that could affect mental health.

**Table 1:** Intervention modules

Objectives	Content	Subjects	Duration	Stage
**Trauma-focused Cognitive Behavioral Intervention Unit**	Provides a supportive environment to help identify child trauma and mental health problem	Psychology teachers; social workers	One week	Beginning of semester
		Head teachers	Three days	
		Administrative staff	One day	
**Recognize common mental health problems**	Help parents and students understand common mental health problems, help parents identify children’s mental health problem	Children/Parents	2 sessions	Beginning of semester
**Cognitive processing of the trauma**	Help students and parents recognize the trauma and its’ characteristics	Children/Parents	2 sessions	2st week at semester
**Affective regulation management, relaxation**	Stress coping skills, resilience, empowerment	Children	3 sessions,	4th week at semester
**Trauma narrative, in vivo mastery of trauma reminders**	Emotional expression and cognition training	Children/Parents	2 sessions	6th week at semester
**Psychoeducation and parenting skills**	Effective parent‒child communication and response during traumatic events	Children/Parents	2 sessions	8th week at semester
**Conjoint child–parent sessions**	Sharing the trauma narrative	Children/Parents	1 session	10th week at semester
**Enhancing future safety and development**	Making a safety plan	Children/Parents	1 session	12th week at semester
**Program evaluation**	Satisfaction evaluation	Children/Parents	1 session	16th week at semester

### Assessments

In this study, we will assess parents, children, and teachers. The assessments were taken before (T1 = one week before intervention), after (T2 = one week after intervention), and follow-up the interventions (T3: six weeks after intervention). To reduce recall bias for health data, we will also combine children’s electronic medical records. The research tools are shown in Table 2. We will use measurement instruments that are well-validated and reliable instruments. The control variables mainly include children’s, parental and teacher’s socio-demographic characteristics. Moderator and mediator variables include children’s resilience, coping methods, family environment, school environment, etc.

**Table 2:** Assessment tools

Category	Variables	Indicator/tools	Data sources
**Outcomes**	PTSS	PTSD Checklist for DSM-5 (PCL-5)	Parents/Child
	Depression	10 Items The Center for Epidemiological Studies-Depression (CESD-10), The Strengths and Difficulties Questionnaire (SDQ)	Child
	Anxiety	Self-Rating Anxiety Scale (SAS)	Child
	Bullying	Delaware bullying victimization scale	Child
**Predictors**	Trauma event	PCL-5 checklist	Child
	Intervention related	Participate, Stratification	Child/teacher
**Confounding**	Child characteristics	Gender, age et al.	Child
	Parent characteristics	Gender, age, marital status et al.	Parent
	Teacher characteristics	Gender, age, teaching background et al.	Teacher
**Moderator/Mediator**	Resilience	14-Item Resilience Scale (RS-14)	Child
	Coping style	Simplified Coping Styles	Child
	Family environment	Family Functioning Style Scale	Child
	School environment	Delaware School Climate scale	Child

### Project activities

Taking advantage of ongoing monitoring and evaluation of the quality of intervention and continuous data collection in the program, this study will achieve the goals and objectives through the following tasks and approaches.

## 1. Design of supportive environment

Initially, our study will contact professional psychology teachers, social workers, head teachers and administrative staff in selected counties and establish long-term partnerships, in order to build a supportive environment supporting conduction of research and protecting child mental health.

## 2. Pilot trails and screening

Before the beginning of the formal study, pilot trails will be conducted to estimate the feasibility of a large RCT of School-based Psychosocial Interventions and improve its detailed design, in the first half-year of 2023. Main task in the second half of 2023 is collecting baseline data before the intervention. Baseline data including basic information (i.e. age, gender) and mental health status of children, their parents and teachers will be assessed via questionnaire surveys. And the core of screening is identifying children’s priority trauma mental health problem and its key roles.

## 3. Trauma recognition and treatment intervention

Intervention will be conducted from the January to December in 2024. The lectures related to mental health will be provided to parents and their children and improve their trauma cognition by school psychology teachers. Next a series of courses and activities will carry out to improve stress coping skills among children. For parent-child sessions, this study will use qualitative interviews methods and hold child-parents trauma narrative discussion to improve effective parent-child communication, reinforcing cognition training.

## 4. Evaluation of intervention and development of recommendations

To evaluate the effectiveness and implementation of the intervention and build a safe school environment for children mental health, our study will collect follow-up data from July 2024 to December 2025, and conduct an effectiveness-implementation study to evaluate the satisfaction and effects of the intervention. Lastly, researchers will write corresponding report and make a school-based health program targeted to physical and mental health of parents and their children.

## 5. Baseline and follow-up questionnaire survey

Questionnaire survey data will be collected by dedicated regional coordinators, graduate students, and undergraduate students. The assessments will be conducted in each cluster at baseline, at the start of the trial, and at subsequent 6-week intervals until the trial ends. All interviewers will receive 2 days of professional training. The training content includes the explanation of the questionnaire, filling instructions and caveats. Prior to the start of the study, each interviewer will conduct a half-day trial interview. According to the trial visit, potential problems appearing in investigation process are explained in a unified manner. All the children, their parents and the school teachers were informed the purpose of this study and signed the informed consent form.

### Statistical methods

All analyses are on an intention-to-treat basis, with all randomized participants included in the analysis and analyzed according to original group assignment regardless of treatment dose received. Descriptive statistics will be used to summarize the characteristics of the participants. Generalized linear mixed models (GLMMs) will be used to evaluate the effectiveness of the intervention on outcomes using a three-level multilevel model. This model includes intervention status, time, and the treatment between intervention and time as fixed effects. Dependency in the repeated measurements within individuals will be modeled using a random intercept and random slope at the participant level. A random intercept at the school level will be used to model dependencies between children within the same school. The interaction between treatment and schools will also be tested to investigate the cluster effects. Confounders will also be included as fixed effects in the model, including gender, age, parent characteristics, and teacher characteristics. The estimated intervention effect will be reported as the mean outcome differences (at T2, T3) and 95% confidence interval (95% CI) between the intervention and control groups. All covariate-by-condition interactions on child mental health will be examined.

## Discussion

This study will identify multilevel factors that influence the implementation of interventions to improve the mental health and well-being of rural children experiencing trauma using a cluster randomized control trial. The findings may provide guidance for potential adjustments to current interventions in rural school settings. Second, the study design will provide a novel model to measure complex interventions in real-world school settings, including measures of effectiveness of implementation strategies for improving rural children’s mental health. Third, the outcomes of this study will generate actionable knowledge where local adaptation is needed for scale-up activities in rural school settings. From a practice perspective, the program supports rural school by easing teachers’ and students’ stress while simultaneously aligning with national strategic priorities to address the growing and critical mental health needs of children. The direct translational research-to-policy implications of this work can revolutionize how mental health intervention programs are delivered in rural primary, middle and high schools. Finally, applying a task-shifting approach will strengthen and expand mental health human capacity among schools, sustainably increase the number of people with access to child mental health prevention, care, and treatment services, enhance the capacity to implement child mental health programmes, and strengthen mental health systems to provide a wide range of quality health-care interventions.

## Feasibility of the study

1. The research content is feasible. This study focuses on the effectiveness of school-based psychosocial interventions on rural children’s traumatic psychological and mental health. Through our previous studies, the types of trauma events and the prevalence and related factors of mental health among rural children were obtained. In this study, children with probable mental health problems were identified, and randomized controlled trials were carried out on the most vulnerable children. Majority evidence has proven the effectiveness of school-based psychosocial interventions for children.
2. Strong experience in field work and good cooperation with the target schools. With the support of the National Natural Science Foundation of China, we conducted an 8-year longitudinal study on the trajectory of mental health symptoms among earthquake survivors. We also conducted a 10-year longitudinal study on mental health problems among migrant and floating children in Beijing, Henan and Gansu. Recently, we completed a baseline survey and assessed mental health problems for more than 20,000 children in Xinjiang. Through these surveys, we have not only established reliable measurements in related research fields, but also accumulated rich experience in field work, and mastered solid data analysis technology, which will ensure the current research in-depth and efficiently.

### Innovation and creativity of the proposed study

This study provides new ideas for mental health promotion and interventions to treat rural children experiencing trauma. This study will apply the task-shifting strategy to the Chinese context. Due to the limited professional mental health human resources in rural areas, mental health resources and services are lacking. The task-shifting strategy provides new ideas for solving the shortage of professional psychotherapy experts in rural China and saves the high cost for families and society. This study focuses on the most vulnerable groups, rural children experiencing trauma in central and western China, who need to be given more attention. To understand the extended effect of school-based interventions, we also include information about child-, school-, and family-related factors. We will work with interdisciplinary teams to identify, prevent and intervene in psychological health in pilot schools.

### Weakness of the proposed study

First, in a real-world randomization control trial, concerns will be raised related to confounding, selection bias, and response rates that may limit the validity and generalizability of the findings. However, this study will use a school-based cluster randomized control design, which is widely used in public health programs. Nevertheless, we will use multicenter cluster randomization and an appropriate analysis plan to address these limitations. Second, acceptance, clarify misconceptions, and cultural compatibility will impact the success of implementation in school children. Thus, the early and consistent involvement of pediatrics, school health workers, teachers, parents and children is the key factor in the development of the mental health intervention manual through rigorous assessment of the risk factors and the knowledge, attitudes and practices associated with mental health.

### Strength of the proposed study

The strengths of this study include the rigorous study design, large sample size, probable high response and follow-up rates and statistical adjustments for school clustering, repeated measures and potential confounders. Cluster randomization can be better tailored for implementation science than individually randomized controlled trials and can potentially reduce treatment contamination between intervention and control groups. Other advantages of using school-based cluster RCTs include implementation convenience, low costs, and improved acceptability and compliance. The school-based psychosocial intervention would be informed by extensive formative research, is culturally appropriate, and expects a statistically significant improvement in rural children’s mental health.

## Data Availability

Please contact the corresponding author for more information.

## Abbreviations

RCT: Randomized Controlled Trial
PTSS: Posttraumatic Stress Symptoms
DALYs: Disability-Adjusted Life-Years
GDP: Gross Domestic Product
TF-CBT: Trauma-Focused Cognitive Behavioral Therapy
PCL-5: PTSD Checklist for DSM-5
GLMMs: Generalized Linear Mixed Models
